# Survival benefit of surgical resection for stage IV gastric cancer: A SEER-based propensity score-matched analysis

**DOI:** 10.3389/fsurg.2022.927030

**Published:** 2022-10-25

**Authors:** Jianhui Sun, Qiong Nan

**Affiliations:** ^1^ Department of Gastroenterology, The First Affiliated Hospital of Kunming Medical University, Kunming, China; ^2^ Yunnan Institute of Digestive Diseases, Kunming, China; ^3^ Graduate School of Kunming Medical University, Kunming, China

**Keywords:** gastric cancer, stage IV, cancer-directed surgery, overall survival rate, SEER

## Abstract

**Background:**

Gastric cancer (GC) is a major malignancy worldwide, and its incidence and mortality rate are increasing year by year. Clinical guidelines mainly use palliative drug combination therapy for stage IV gastric cancer. In accordance with some small sample studies, surgery can prolong survival. There is no uniform treatment plan for stage IV gastric cancer. This study focused on collecting evidence of the survival benefit of cancer-directed surgery (CDS) for patients with stage IV gastric cancer by analyzing data from a large sample.

**Methods:**

Data on patients with stage IV gastric cancer diagnosed between 2010 and 2015 was extracted and divided into CDS and no-CDS groups using the large dataset in the Surveillance, Epidemiology, and End Results (SEER) database. With bias between the two groups minimized by propensity score matching (PSM), the prognostic role of CDS was studied by the Cox proportional risk model and Kaplan-Meier.

**Results:**

A total of 6,284 patients with stage IV gastric cancer were included, including 514 patients with CDS who were matched with no-CDS patients according to propensity score (1:1), resulting in the inclusion of 432 patients each in the CDS and no-CDS groups. The results showed that CDS appeared to prolong the median survival time for stage IV gastric cancer (from 6 months to 10 months). Multifactorial analysis showed that poorly differentiated tumors (grades III-IV) significantly affected patient survival, and chemotherapy was a protective prognostic factor.

**Conclusion:**

The findings support that CDS can provide a survival benefit for stage IV gastric cancer. However, a combination of age, underlying physical status, tumor histology, and metastatic status should be considered when making decisions about CDS, which will aid in clinical decision-making.

## Introduction

Gastric cancer (GC) is a major malignancy worldwide, ranking fifth in terms of incidence and fourth in terms of mortality (1). In China, gastric cancer has the second-highest incidence and fatality rates of all malignant tumors (2). According to the bulk of gastric cancer cases, progressive gastric cancer accounts for more than 90%, of which unresectable gastric cancer accounts for about 10% (3). By combining surgery, radiotherapy, and adjuvant chemotherapy, the 5-year survival rate for early stomach cancer can reach 95%. However, according to statistics, the 5-year survival rate for progressive gastric cancer is still less than 50%, and between 80% and 90% of gastric cancers develop into advanced stages and become incurable or recur within 5 years following surgery (4). Due to the insidious nature of gastric cancer and the invasive biological properties of cancer, the majority of patients have distant metastasis by the time they are detected. According to the AJCC cancer staging criteria, stage IV gastric cancer is classified as locally progressive gastric cancer that invades adjacent organs or gastric cancer with distant metastases (5). Traditionally, this category of patients was believed to be incurable, and therapy consisted primarily of a combination of palliative medications. The National Comprehensive Cancer Network (NCCN) and the European Society for Medical Oncology (ESMO) guidelines for the treatment of advanced gastric cancer both advocate chemotherapy or combination therapy for advanced gastric cancer (6, 7). Additionally, the Japanese guidelines encourage the use of chemotherapy or combination therapy. Also in the Japanese guidelines, surgery is specifically mentioned as a palliative treatment aimed at managing symptoms such as obstruction and bleeding (8). In addition, stenting, radiotherapy, and symptomatic treatment should also be considered (9). Surgical resection for the benefit of patients is becoming achievable with the advancement of surgical procedures and mastery of laparoscopic techniques. Several studies have demonstrated that surgery can be conducted more safely, with a lower surgical risk, and may result in an increase in survival (10–13). Nevertheless, the decision to undergo cancer-directed surgery (CDS) for patients with advanced tumors is sometimes based on the surgeon's own choice (14). Although standard recommendations do not support the surgical excision of stage IV gastric cancer, in patients with advanced gastric cancer who underwent combined gastrectomy and hepatectomy, the median overall survival (OS) for the liver was 21 months, as reported in a systematic review, suggesting that surgical resection is beneficial in these patients (15). Eight studies compared surgical resection with other palliative treatments. Subgroup analysis found that patients who underwent liver resection had improved survival and a 20% lower risk of overtime death. To some extent, this also clarifies the potential beneficial role of surgery in the treatment of patients with metastatic gastric cancer (16). In the case of liver metastases from gastric cancer, C-GCLM staging type I and some type II are feasible for comprehensive surgery-centered treatment, and it is noted that resection of the primary site and metastases can increase the overall 5-year survival rate of patients with liver metastases from gastric cancer to more than 20% under strict screening of the patient population (17). Yu et al. (18) conducted a retrospective analysis of the treatment of 132 patients with concurrent liver metastases. The results showed that R0 resection significantly prolonged survival time (33.6 months vs. 12 months). (33.6 months vs. 12.4 months, *P* < 0.001). Yu et al. (19) showed that the prognosis of the group receiving systemic therapy + resection was better than that of the palliative chemotherapy group (21.1 months vs. 10.8 months, *P* = 0.002) in the treatment of peritoneal metastases from gastric cancer with a primary exploration PCI < 20, and found that patients with a secondary laparoscopic exploration PCI < 6 after systemic therapy had a better prognosis. In summary, many studies have indicated that CDS is most advantageous for patients with stage IV gastric cancer who have received translational therapy (systemic chemotherapy and local radiotherapy). The selection of patients for surgery after chemotherapy for stage IV gastric cancer depends largely on the degree of response to chemotherapy, and a good response to chemotherapy and the ability to achieve R0 resection are the most important screening indicators for surgical treatment. Therefore, some advanced gastric cancers still have certain surgical value, and actively choosing the appropriate timing and surgical method can help prolong survival and improve prognosis. As a result, our work was confirmed further by extracting large-sample, multicenter data from the SEER database, which revealed a correlation between CDS and increased overall survival in patients with stage IV gastric cancer. However, due to the fact that CDS is not a guideline-recommended standard of care, surgeons' decisions to perform surgery are highly selective. This potential results in a non-random bias in overall survival for patients who produce CDS compared to those who do not. This raises many critical difficulties. First, there is a dearth of research regarding the longevity of individuals with stage IV gastric cancer following surgical resection. Second, there is a dearth of medical evidence or appropriate criteria to assist surgeons in identifying individuals who are candidates for surgical resection. The ability of surgical resection to provide a survival benefit to patients has not been well studied. To address this uncertainty, based on the SEER database, this study controls for potential confounders by using propensity score matching (PSM) to verify whether there is an improvement in survival in patients with stage IV gastric cancer treated with CDS. PSM was combined with other prognostic factor indicators to provide a more reliable estimate of survival for CDS patients and ultimately guide clinical decision-making.

## Patients and methods

### Patients

Between 2010 and 2015, data on patients with stage IV gastric cancer were extracted from the SEER database. The following criteria were used to determine inclusion and exclusion: (1) patients with pathological histologically confirmed primary gastric adenocarcinoma and tumor M stage M1; (2) demographic information including age, race, gender, and marital status included; (3) clinicopathological information including primary site, differentiation level, T stage, and N stage included. (4) Patients with incomplete demographic and clinicopathological information were excluded. The final 6,284 patients diagnosed with stage IV gastric cancer were included in this research, and the patients were divided into those with cancer treated with CDS (CDS group) and those not treated with CDS (no-CDS group), including 514 patients with CDS who were propensity score-matched (1:1) to those with no-CDS, and 432 patients each in the CDS and no-CDS groups were finally included. This was ultimately used to gather evidence for the benefit of CDS for stage IV gastric cancer.

## Data collection

Parameters such as age, race, gender, marital status, tumor primary site, differentiation grade, T stage, N stage, chemotherapy, and overall survival were selected for this study, and due to the fact that the SEER database no longer contains information on tumor T stage, tumor N stage, or chemotherapy after 2015, we only selected data before 2015.

## Statistical analysis

### Propensity score matching (PSM)

Subjects were matched by propensity score (1:1), a process that reduces selective bias for specific patients treated with CDS, and then compared survival outcomes for patients in the matched CDS and no-CDS groups. Notably, validation of PSM was achieved by comparing each observed variable in the CDS and no-CDS groups before and after PSM. *χ*2 test was used to compare categorical variables, while the unpaired Student's t-test was used to compare continuous variables.

### Survival analysis

The study was statistically analyzed using R software (version 4.1.2). A *P* value of 0.05 was considered statistically significant. The log-rank test was used to compare the median survival rates of CDS groups. The Kaplan-Meier method was used to calculate overall survival. Models were also screened automatically using a stepwise method and AIC (Akaike Information Criterion) was calculated for each generated model, AIC values were used to select the 95% confidence set, which may contain the best approximation model for all the data considered. Moreover, we averaged hazard ratio estimates for CDS and other predictors at 95% confidence intervals, which were used to infer prognostic factors for survival.

## Results

### Baseline characteristics of study subjects and propensity score matching

A total of 6,284 patients with stage IV gastric cancer were included, of whom 514 received CDS and 5,770 did not. The most common tumor primary sites were cardia and fundus (27.2% in the CDS group and 47.8% in the no-CDS group), the most common differentiation grade was grade III (69.1% in the CDS group and 52.8% in the no-CDS group), the most common T stage was T1 (9.3% in the CDS group and 20.5% in the no-CDS group), and the most common N stage was N1 (27.2% in the CDS group and 37.9% in the no-CDS group), respectively. For the clinical characteristics of stage IV gastric cancer patients such as age, race, gender, and marital status, age was concentrated above 65 years (51.9% in the CDS group and 56.2% in the no-CDS group), males were higher than females (67.9% in the CDS group and 70.1% in the no-CDS group), racial groups were more common in whites (60.9% in the CDS group and 57.6% in the no-CDS group), and marital status was mostly seen in married (66.3% in the CDS group, 75.0% in the no-CDS group). In addition, the CDS group had a considerably longer mean survival duration than the no-CDS group (16 months in the CDS group, 8.64 months in the no-CDS group) (Table 1).

**Table 1:** Baseline characteristics before propensity matching scores, showing statistical comparisons between the CDS and no-CDS groups.

	CDS	No-CDS	Overall	*χ* ^2^	*p*
	(*N* = 514)	(*N* = 5770)	(*N* = 6284)		
Age				3.521	0.172
≤49	61 (11.9%)	644 (11.2%)	705 (11.2%)		
50-64	186 (36.2%)	1,884 (32.7%)	2,070 (32.9%)		
≥65	267 (51.9%)	3,242 (56.2%)	3,509 (55.8%)		
Sex				0.972	0.324
Female	165 (32.1%)	1,726 (29.9%)	1,891 (30.1%)		
Male	349 (67.9%)	4,044 (70.1%)	4,393 (69.9%)		
Race				26.668	<0.001
White	341 (66.3%)	4,326 (75.0%)	4,667 (74.3%)		
Black	71 (13.8%)	739 (12.8%)	810 (12.9%)		
Other	100 (19.5%)	687 (11.9%)	787 (12.5%)		
Unknown	2 (0.4%)	18 (0.3%)	20 (0.3%)		
Marital status				6.569	0.161
Divorced	41 (8.0%)	503 (8.7%)	544 (8.7%)		
Married	313 (60.9%)	3,322 (57.6%)	3,635 (57.8%)		
Single	61 (11.9%)	893 (15.5%)	954 (15.2%)		
Widowed	59 (11.5%)	680 (11.8%)	739 (11.8%)		
Unknown	40 (7.8%)	372 (6.4%)	412 (6.6%)		
Primary Site				148.950	<0.001
Body of stomach	40 (7.8%)	488 (8.5%)	528 (8.4%)		
Overlapping lesion of stomach	48 (9.3%)	378 (6.6%)	426 (6.8%)		
Stomach	75 (14.6%)	953 (16.5%)	1,028 (16.4%)		
Cardia and fundus of stomach	140 (27.2%)	2,756 (47.8%)	2,896 (46.1%)		
Gastric antrum and pylorus	149 (29.0%)	730 (12.7%)	879 (14.0%)		
Greater and lesser curvature	62 (12.1%)	465 (8.1%)	527 (8.4%)		
Grade				69.774	<0.001
Grade I	4 (0.8%)	125 (2.2%)	129 (2.1%)		
Grade II	128 (24.9%)	1,342 (23.3%)	1,470 (23.4%)		
Grade III	318 (61.9%)	3,046 (52.8%)	3,364 (53.5%)		
Grade IV	17 (3.3%)	52 (0.9%)	69 (1.1%)		
Unknown	47 (9.1%)	1,205 (20.9%)	1,252 (19.9%)		
T				543.500	<0.001
T0	0 (0%)	33 (0.6%)	33 (0.5%)		
T1	48 (9.3%)	1,185 (20.5%)	1,233 (19.6%)		
T2	28 (5.4%)	174 (3.0%)	202 (3.2%)		
T3	173 (33.7%)	730 (12.7%)	903 (14.4%)		
T4	216 (42.0%)	866 (15.0%)	1,082 (17.2%)		
Tx	49 (9.5%)	2,782 (48.2%)	2,831 (45.1%)		
N				849.840	<0.001
N0	101 (19.6%)	2,036 (35.3%)	2,137 (34.0%)		
N1	140 (27.2%)	2,189 (37.9%)	2,329 (37.1%)		
N2	110 (21.4%)	258 (4.5%)	368 (5.9%)		
N3	134 (26.1%)	170 (2.9%)	304 (4.8%)		
Nx	29 (5.6%)	1,117 (19.4%)	1,146 (18.2%)		
Chemotherapy				0.429	0.512
No	221 (43.0%)	2,389 (41.4%)	2,610 (41.5%)		
Yes	293 (57.0%)	3,381 (58.6%)	3,674 (58.5%)		
Survival months					
Mean (SD)	16.0 (19.4)	8.64 (12.6)	-	329.13	<0.001
Median [Min, Max]	9.00 [0, 103]	4.00 [0, 107]	-		

**Figure 1:**
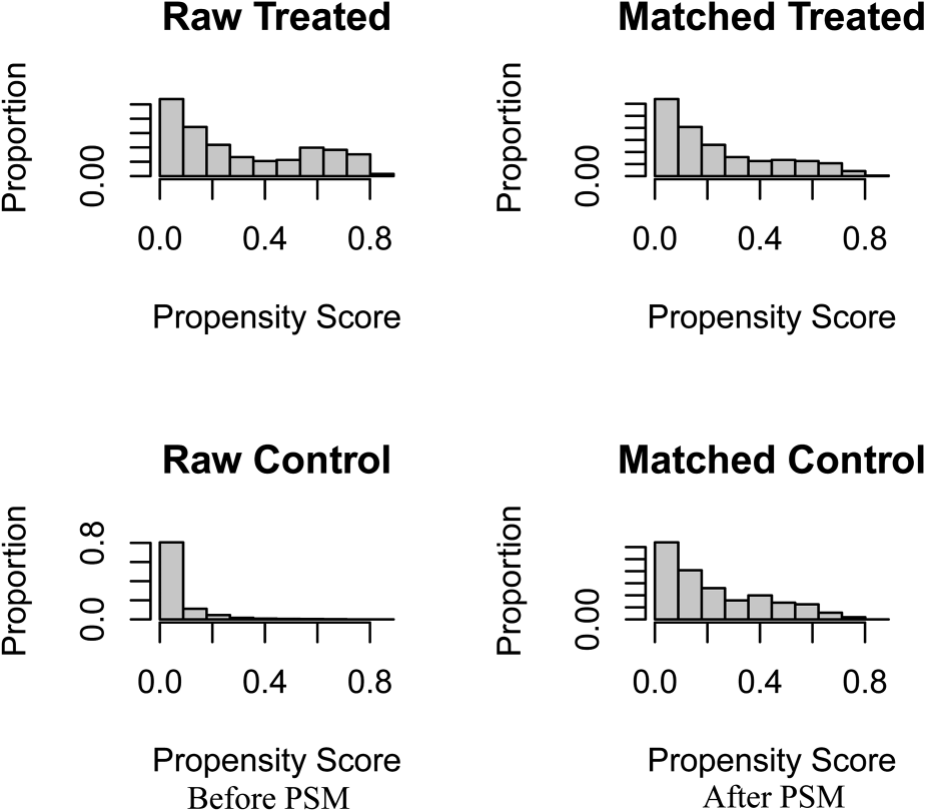
Before and after changes in propensity score matching between the CDS and No-CDS groups. CDS: cancer-directed surgery, PSM: propensity score matching.

After matching patients in the CDS and no-CDS groups 1:1, there were 432 patients in each of the two groups, with *P* > 0.05 for each variable after propensity score matching (Table 2, Figure 1). Prior to PSM, the data showed significant differences in baseline characteristics between the CDS and no-CDS groups for various variable parameters. After PSM, there were no significant differences between the two groups on multiple variables (Table 2).

**Table 2:** Baseline characteristics after propensity matching scores, showing statistical comparisons between the CDS and no-CDS groups.

	CDS	No-CDS	Overall	χ^2^	*p*
	(*N* = 432)	(*N* = 432)	(*N* = 864)		
Age				3.119	0.210
≤49	50 (11.6%)	35 (8.1%)	85 (9.8%)		
50–64	153 (35.4%)	165 (38.2%)	318 (36.8%)		
≥65	229 (53.0%)	232 (53.7%)	461 (53.4%)		
Sex				0.551	0.458
Female	135 (31.3%)	124 (28.7%)	259 (30.0%)		
Male	297 (68.8%)	308 (71.3%)	605 (70.0%)		
Race				1.443	0.696
White	295 (68.3%)	311 (72.0%)	606 (70.1%)		
Black	59 (13.7%)	51 (11.8%)	110 (12.7%)		
Other	77 (17.8%)	69 (16.0%)	146 (16.9%)		
Unknown	1 (0.2%)	1 (0.2%)	2 (0.2%)		
Marital status				2.566	0.633
Divorced	31 (7.2%)	31 (7.2%)	62 (7.2%)		
Married	262 (60.6%)	266 (61.6%)	528 (61.1%)		
Single	57 (13.2%)	43 (10.0%)	100 (11.6%)		
Widowed	48 (11.1%)	54 (12.5%)	102 (11.8%)		
Unknown	34 (7.9%)	38 (8.8%)	72 (8.3%)		
Primary Site				2.975	0.704
Body of stomach	36 (8.3%)	28 (6.5%)	64 (7.4%)		
Overlapping lesion of stomach	39 (9.0%)	36 (8.3%)	75 (8.7%)		
Stomach, NOS	65 (15.0%)	72 (16.7%)	137 (15.9%)		
Cardia and fundus of stomach	139 (32.2%)	151 (35.0%)	290 (33.6%)		
Gastric antrum and pylorus	97 (22.5%)	99 (22.9%)	196 (22.7%)		
Greater and lesser curvature	56 (13.0%)	46 (10.6%)	102 (11.8%)		
Grade				2.969	0.563
Grade I	4 (0.9%)	2 (0.5%)	6 (0.7%)		
Grade II	116 (26.9%)	105 (24.3%)	221 (25.6%)		
Grade III	259 (60.0%)	258 (59.7%)	517 (59.8%)		
Grade IV	6 (1.4%)	9 (2.1%)	15 (1.7%)		
Unknown	47 (10.9%)	58 (13.4%)	105 (12.2%)		
T				4.818	0.307
T1	48 (11.1%)	50 (11.6%)	98 (11.3%)		
T2	28 (6.5%)	24 (5.6%)	52 (6.0%)		
T3	144 (33.3%)	165 (38.2%)	309 (35.8%)		
T4	163 (37.7%)	136 (31.5%)	299 (34.6%)		
Tx	49 (11.3%)	57 (13.2%)	106 (12.3%)		
N				4.524	0.340
N0	101 (23.4%)	78 (18.1%)	179 (20.7%)		
N1	140 (32.4%)	155 (35.9%)	295 (34.1%)		
N2	88 (20.4%)	84 (19.4%)	172 (19.9%)		
N3	74 (17.1%)	81 (18.8%)	155 (17.9%)		
Nx	29 (6.7%)	34 (7.9%)	63 (7.3%)		
Chemotherapy				3.351	0.067
No	174 (40.3%)	147 (34.0%)	321 (37.2%)		
Yes	258 (59.7%)	285 (66.0%)	543 (62.8%)		
Survival months					
Mean (SD)	16.7 (19.7)	10.6 (15.2)		102.65	<0.001
Median [Min, Max]	10.0 [0, 103]	5.00 [0, 103]			

## Survival outcome after propensity score matching

**Figure 2:**
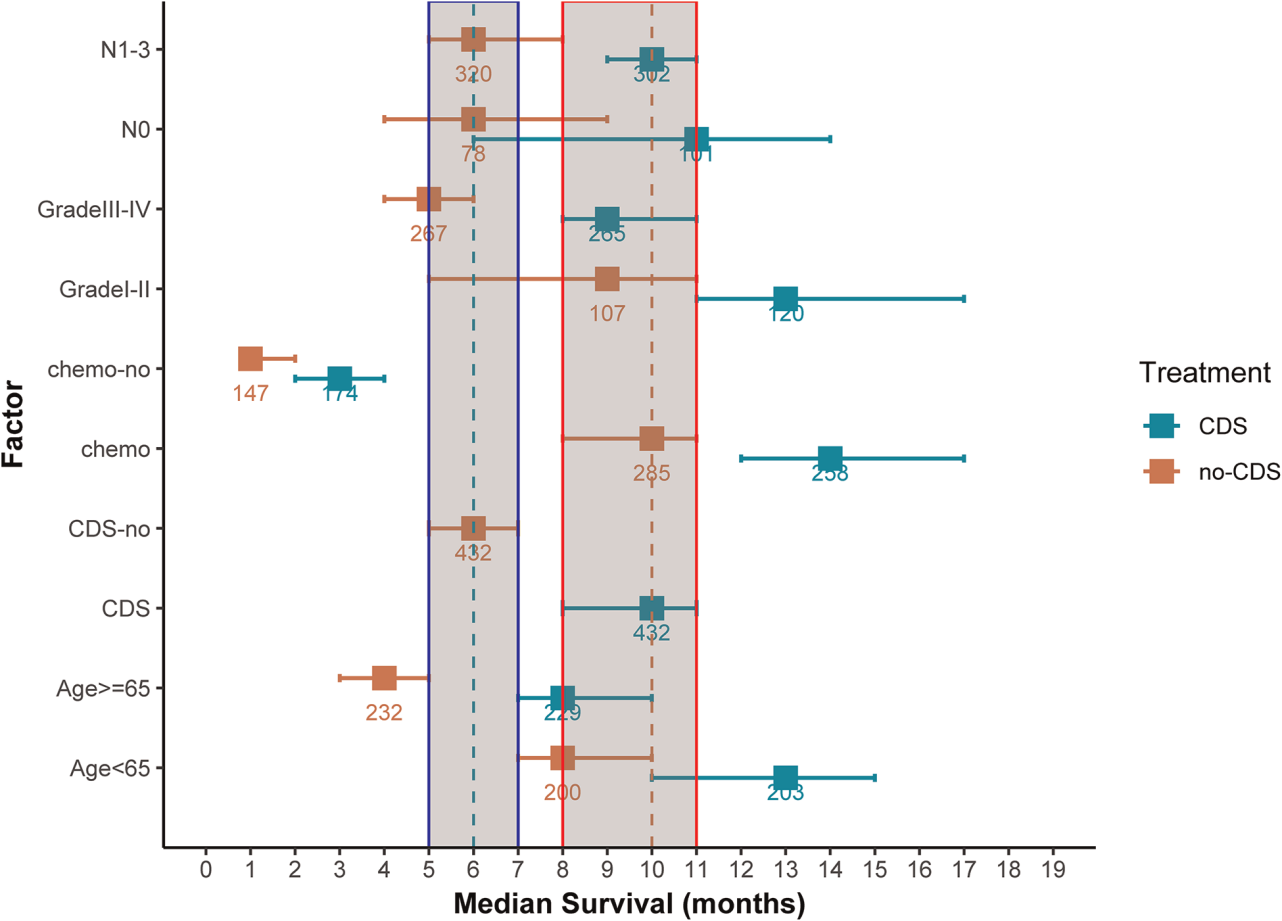
Median overall survival (months) ± 95% confidence interval for patients in the CDS and No-CDS groups. The dashed and shaded areas cover the median and confidence intervals for the total sample. Numbers are sample sizes. For ease of reading and understanding, certain factor levels have been removed and results are given for the total sample and for meaningful prognostic factors. CDS: cancer-guided surgery, Grade: tumor differentiation grade, N: tumor stage N, chemo: chemotherapy.

**Figure 3:**
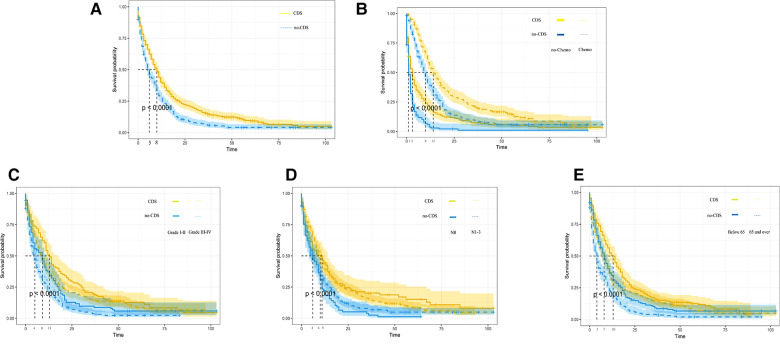
Kaplan-Meier overall survival (OS) estimates and 95% confidence intervals for patients in the CDS and no-CDS groups. (**A**) In the total sample, (**B**) Chemotherapy, (**C**) Grade, (**D**) N-stage, and (**E**) age. CDS: cancer-guided surgery.

As shown in Figures 2, 3A, comparing the two groups after PSM (432 patients each), the median survival was higher in CDS patients (8–11 months) than in non-CDS patients (5–7 months). In addition, the 12-month predicted survival rate was 1.47 times higher for CDS patients than for non-CDS patients (CDS [95CI]: 0.358–0.451; non-CDS [95CI]: 0.2337–0.3191), and the 24-month predicted survival rate was 2.17 times higher for CDS patients than for non-CDS patients (CDS [95CI]: 0.189–0.269 CDS [95CI]: 0. 0786–0.1381), and the 36-month predicted survival rate for CDS patients was 2.44 times that of non-CDS patients (CDS [95CI]: 0.124–0.193; non-CDS [95CI]: 0.0437–0.0923) (Figure 3A). there was a significant improvement in survival for CDS patients (mean survival in the CDS group survival was 16.7 months in the CDS group and 10.6 months in the non-CDS group) (Table 2). A relatively close model (AIC = 9147.59) was finally identified using a stepwise method to automatically screen the model. This model suggested that factors that could predict survival included (1) age, (2) race, (3) grade of differentiation, (4) tumor T stage, (5) chemotherapy, and (6) CDS (Table 3).

**Table 3:** Stepwise regression analysis method for automated model screening.

	HR	95%CI	*P*
Age			
<65	Reference		
≥65	1.14534	1.01315876–1.2947705	0.069
Race			
Black	Reference		
White	1.15323	0.96327929–1.3806284	0.193
Other	1.06197	0.85117918–1.3249732	0.655
Unknown	0.15777	0.02983461–0.8343466	0.068
Grade			
Grade I-II	Reference		
Grade III-IV	1.42907	1.24228609–1.6439408	<0.001
Unknown	1.21551	0.98108638–1.5059437	0.134
T			
T1	Reference		
T2	0.84571	0.62794846–1.1389890	0.356
T3	0.86839	0.70968537–1.0625911	0.250
T4	1.13828	0.92875278–1.3950809	0.295
Tx/NA	1.34726	1.05125127–1.7266097	0.048
Chemotherapy			
No	Reference		
Yes	0.33161	0.29021219–0.3789054	< 0.001
Surgery			
CDS	Reference		
no-CDS	1.78204	1.57616003–2.0148153	< 0.001

HR, Hazard ratio; CI, Confidence interval.

**Figure 4:**
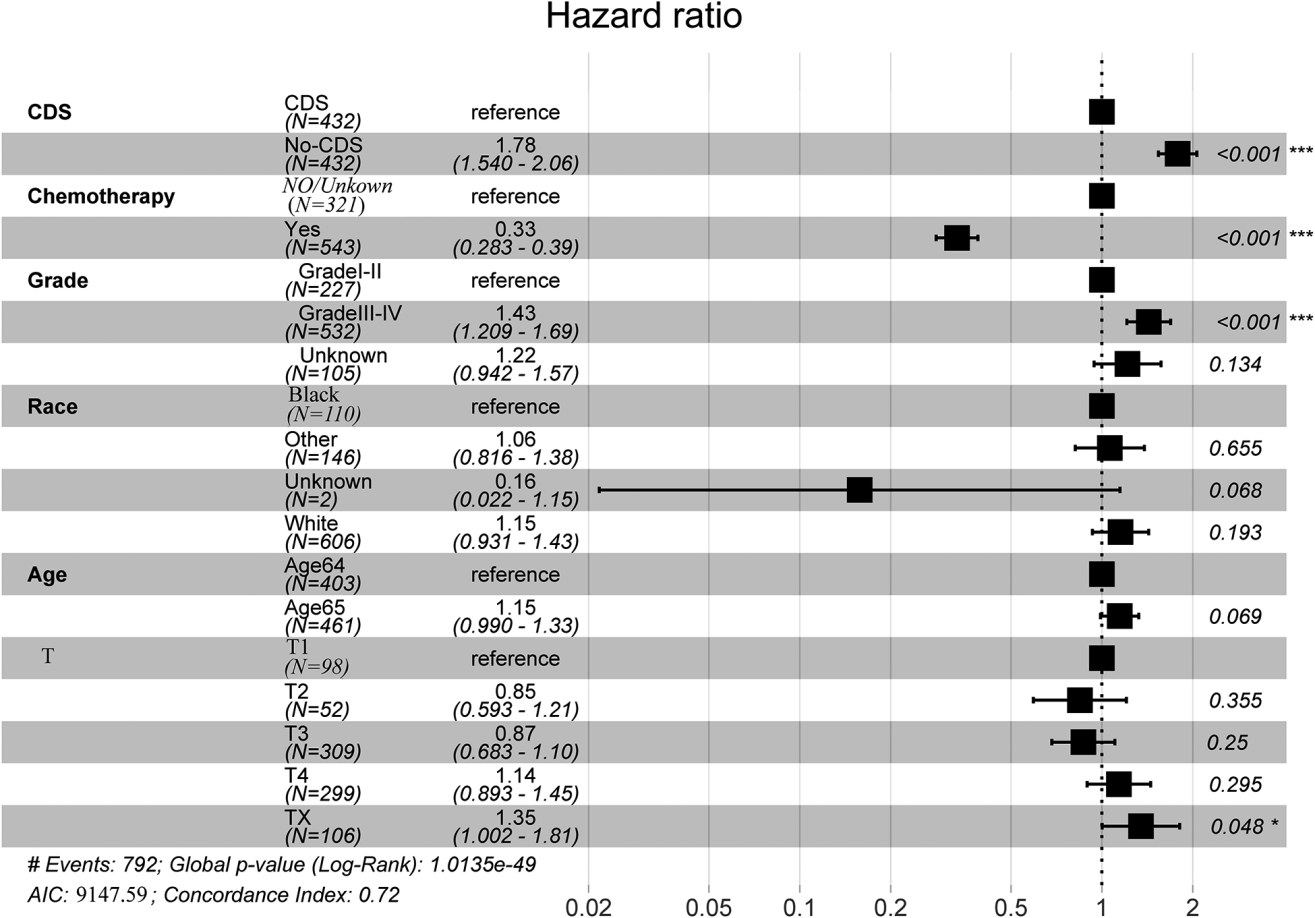
Full model average Cox proportional hazard ratios with 95% confidence intervals. There is a dashed line indicating the equivalent hazard ratio (HR = 1) (Table 3).

In addition, we finally obtained risk ratio estimates for each factor in the model by calculating the estimates for each model that was in the mean confidence set. The results showed that CDS was a factor of significant value in the model (Table 3). Additionally, our findings indicated that patients with CDS had a greater survival rate than those without (Figure 4), and among the other factors analyzed, receiving chemotherapy also significantly improved the survival rate of patients with advanced gastric cancer, and tumor hypofractionation (grade III-IV) similarly affected the survival rate of patients (Table 3, Figures 3B–E, 4). It is worth mentioning that race, age, and tumor T-stage were also included in this model, but did not seem to be more significant in terms of predictive accuracy than CDS, chemotherapy, and differentiation grade.

## Discussion

This study could show by comparing matched cohorts in the SEER database that overall survival was significantly longer in patients with stage IV gastric cancer treated with CDS than in those not treated with CDS. The analysis also showed that chemotherapy and the degree of tumor differentiation were meaningful prognostic indicators. These results suggest that CDS is most effective in treating patients with stage IV gastric cancer who have received chemotherapy and have a good degree of tumor differentiation. However, CDS may also provide meaningful survival improvement for patients with a poor prognosis. Therefore, age, underlying physical condition, tumor histology, and metastatic status, all of these conditions should be taken into consideration in clinical practice.

GC has a high incidence, insidious onset, and lacks obvious or characteristic clinical manifestations in early stages. In China, the detection rate of early gastric cancer is much lower than that of Japan and Korea due to the lack of popularity of gastroscopy, which results in most patients being diagnosed with gastric cancer at progressive or advanced stages (stage IV). Early or progressive gastric cancer can be treated surgically with R0 resection and associated site lymph node dissection to achieve a relatively good prognosis. However, for patients with stage IV gastric cancer, the 5-year survival rate is only 4% (5). According to a Japanese survey, the 5-year survival rate of stage IV gastric cancer can be increased to 16.4% by surgical resection or chemotherapy interventions (20). Therefore, it is critical to study how surgical resection affects the survival and prognosis of patients with stage IV gastric cancer and to develop a more systematic and beneficial treatment plan.

In conventional wisdom, numerous researchers feel that surgical excision of stage IV gastric cancer does not improve overall survival. Because advanced gastric cancer is inherently more difficult to resect surgically than early or progressive gastric cancer, and because surgery is more time-consuming and cancer patients are in long-term negative nitrogen balance, the benefit of surgery in the treatment of stage IV gastric cancer is not clear. Based on the MAGIC and FNCLCC/FFCD9703 studies, in the majority of European countries, chemotherapy is the conventional treatment technique for progressive gastric cancer (21). The REGATTA study further rejected the use of palliative surgery in the initial treatment of advanced gastric cancer (22). AL-BATRAN et al. (23) advocated that chemotherapy before considering surgery might benefit patient survival. The three major guidelines of NCCN, ESMO, and JGCA also recommend unless serious complications such as bleeding and obstruction occur, which seriously threaten patients' lives. Because the probability of intraoperative and postoperative complications is higher than that of conventional surgery in general, and resection does not significantly prolong patient survival, but rather affects the subsequent quality of life, Fujitani et al. concluded that non-radical surgery decreases chemotherapy adherence without any prognostic benefit (22).

On the contrary, in recent years, palliative primary resection for stage IV gastric cancer has gradually become a consensus, especially for younger patients with more differentiated tumor cells and lower tumor grade. Patients with stage IV gastric cancer benefit from palliative surgical resection (24–27). Min et al. (28) concluded that in patients with stage IV gastric cancer, radical gastrectomy may be an option. In certain patients with stage IV gastric cancer, laparoscopic gastrectomy is safe and viable. Sun et al. (13) conducted a Meta-analysis of 14 publications containing 3,003 cases and found that palliative resection in patients with stage IV gastric cancer where radical resection was not possible improved long-term survival, especially in stage M1 gastric cancer. A multi-institutional analysis in China suggested that patients with progressing gastric cancer may benefit from radical surgical resection (29). In addition, surgical resection may reduce some acute complications during chemotherapy, such as bleeding, obstruction, and carcinoid syndrome. These acute complications also require urgent surgical treatment when they occur. However, without adequate preoperative preparation, the incidence of postoperative complications will increase, which in turn will reduce the quality of patients' survival after surgery and even accelerate their death. For early-stage gastric cancer and progressive gastric cancer, R0 resection can often be achieved through surgical resection, i.e., “no evidence of disease (NED”),”, is the principle of GC surgical treatment. However, whether the patient can be safely transitioned and whether the primary tumor can meet the criteria of R0 resection at the time of surgical resection is an issue that should be carefully considered by the surgeons before surgery (30). Seo et al. showed the benefit of surgery, with median survival times of 41.3 months and 21.2 months in patients undergoing translational surgery after chemotherapy for R0 and R1–2 resections, respectively (31). These data imply that R status may have an effect on the prognosis of stage IV gastric cancer patients undergoing conversion surgery. Overall survival was considerably longer in the CDS group than in the no-CDS group for patients with stage IV gastric cancer, which was also better validated in the matched cohort in the SEER database. In addition, differentiation grade and chemotherapy were meaningful prognostic factors. In this study, the findings showed that CDS was most effective when patients received chemotherapy and had well-differentiated tumors.

We believe that the improvement in overall survival of patients with stage IV gastric cancer following surgical resection is due to several factors: first, surgical resection reduces the tumor burden and restores some immune capacity to the patient, even in metastatic lesions (32). Second, after tumor resection, chemotherapy is more effective in people with stage IV gastric cancer following surgery, resulting in improved survival rates. Finally, in patients with stage IV gastric cancer, surgical resection decreases the probability of acute complications such as bleeding, blockage, and perforation. It is worth mentioning that an inappropriate surgical approach may accelerate the medical spread of tumors and postoperative recurrence and metastasis (33).

In recent years, an increasing number of scholars have provided new insights. For example, based on the successful practice of conversion therapy in liver metastases from colorectal cancer, conversion therapy has been attempted in stage IV GC (34, 35). The study by Cascinu et al. (36) included 82 patients with stage IV GC, 37 of whom underwent post-transformation surgery, and at the end of the 48-month interim follow-up, the survival rate of the operated patients was 68%, and the median survival was significantly better than that of the non-operated patients. While Yoshida et al. (37) proposed a new idea of Yoshida staging of advanced gastric cancer based on the biological behavior of gastric cancer, scholars tried to explore individualized treatment of gastric cancer patients in terms of molecular staging, multi-omics, and artificial intelligence big data analysis. We believe that surgeons or clinicians should fully consider the complementary nature of surgery and systemic therapy, as well as the combination of surgery and novel adjuvant chemotherapy before making a decision. In addition, the clinical characteristics of patients, tumor biology, and whether or not they receive chemotherapy can affect the overall survival rate.

In conclusion, the treatment of stage IV gastric cancer is a difficult clinical problem. Gastric cancer has multiple metastases, and there are more adjacent organs around the stomach with abundant blood vessels, which undoubtedly adds a higher degree of difficulty to surgical resection. Secondly, there is no unanimous consensus on whether surgical treatment has a clear improvement on the overall survival, survival rate, and quality of life of patients. Thirdly, the tension between doctors and patients makes it necessary for surgeons to be more cautious when choosing surgical treatment. Ultimately, only a fraction of patients were treated with CDS due to subjective or objective factors, while chemotherapy remains the mainstay of stage IV GC for a significant proportion of patients, especially for those with a poor physical foundation, multiple underlying diseases, and advanced age. More randomized controlled studies are still needed to verify which surgeries will be beneficial in the future for patients with stage IV GC.

There are also some obvious limitations and shortcomings in this study: (1) The data analyzed in this study were all derived from the SEER database, i.e., the data resolution was low for clinically significant variables that may be critical to the overall survival of patients with stage IV GC treated with CDS. Moreover, we cannot obtain detailed information about patients from them, such as their underlying disease status, whether they have a family history of tumors, their preoperative or postoperative chemotherapy regimen and chemotherapy cycles, specific surgical procedures, and postoperative quality of life. (2) Usually, in clinical work, patients who choose surgical resection are mostly with less underlying disease and better health status, so there is some selective bias in this study. However, these limitations can only be addressed by the randomized controlled trial method. (3) The SEER database included mainly Americans, while malignant tumors often have racial differences in metastasis and survival in different organs, and whether the study results are applicable to other countries or ethnic groups remains to be studied in depth. (4) This study screened data from 2010 to 2015, but the current international guidelines for stage IV GC are still dominated by chemotherapy, and more in-depth studies are needed in the future to determine whether surgery is appropriate and the survival benefit brought by surgical treatment to patients. Therefore, the results of this study are not representative of survival in all stage IV gastric cancers, and caution is still needed in interpreting these results. However, the SEER database includes a broad population of 30% of the US population, and the results of clinical studies will become increasingly convincing in the future as the included population continues to expand.

## Conclusion

Although the benefits of CDS in malignancies are well recognized, the value of CDS in stage IV gastric cancer remains highly controversial. Different scholars also hold different attitudes regarding the survival benefit of CDS in patients with stage IV gastric cancer. Our study provides evidence for the possible survival benefit of CDS for patients with stage IV gastric cancer. However, given the aforementioned shortcomings and certain limitations of this paper, it is important to explore the multidisciplinary and multimodal approach of CDS in patients with stage IV gastric cancer and to combine it with radiotherapy, chemotherapy, targeted drugs, and immunotherapy to develop a personalized treatment plan based on precise classification in order to possibly help patients with advanced gastric cancer to obtain the maximum survival and quality of life. In the future, large sample, multicenter randomized controlled trials and evidence-based medical studies are still needed to validate and ultimately help clinical decision making.

## Data Availability

The SEER Dataset Repository (https://seer.cancer.gov/) contains datasets from the SEER database that were created and/or processed for this study currently available.
