# Supplementary material for: Survival benefit of surgical resection for stage IV gastric cancer: A SEER-based propensity score-matched analysis
Source: Front Surg. 2022 Oct 25;9:927030. doi: 10.3389/fsurg.2022.927030 (PMC9640680; doi:10.3389/fsurg.2022.927030)

Figure 1. Before and After changes in propensity score matching between the CDS and No-CDS groups. CDS: cancer-directed surgery, PSM: propensity score matching.

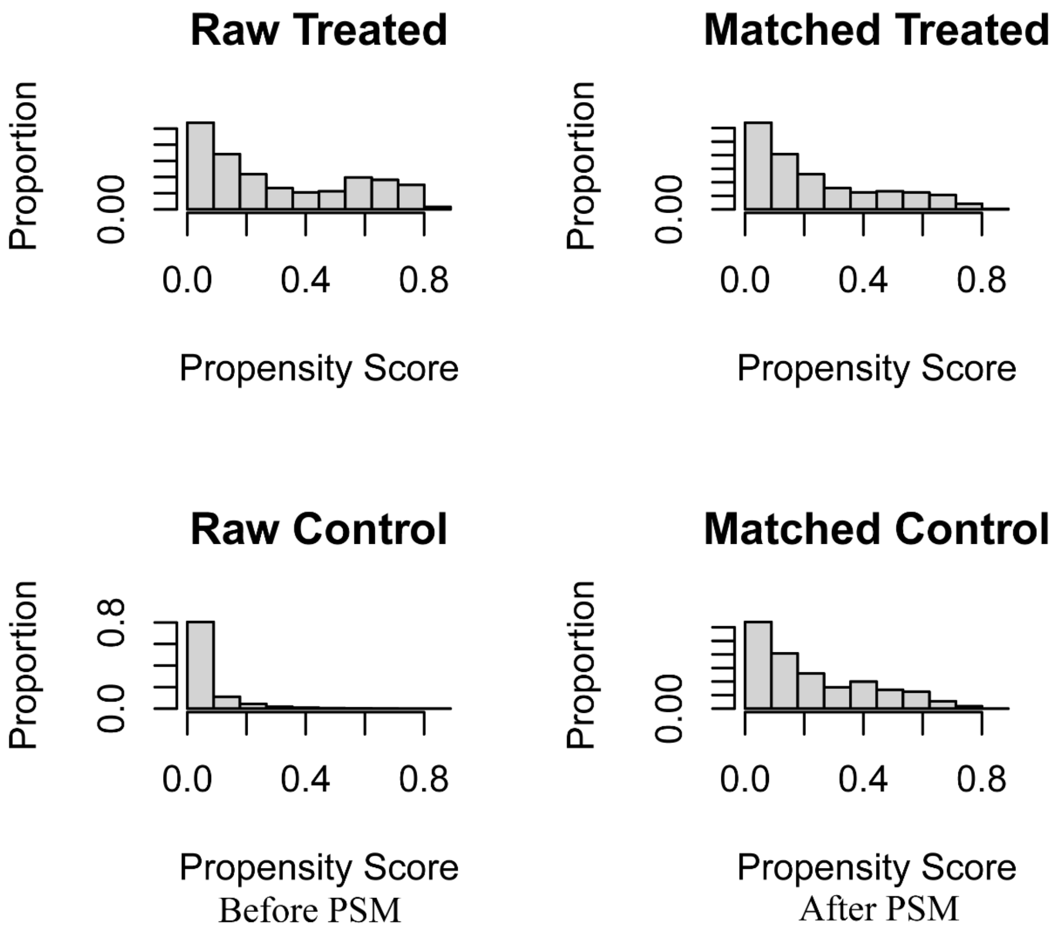

Figure 2. Median overall survival (months) ± 95% confidence interval for patients in the CDS and No-CDS groups. The dashed and shaded areas cover the median and confidence intervals for the total sample. Numbers are sample sizes. For ease of reading and understanding, certain factor levels have been removed and results are given for the total sample and for meaningful prognostic factors. CDS: cancer-guided surgery, Grade: tumor differentiation grade, N: tumor stage N, chemo: chemotherapy.

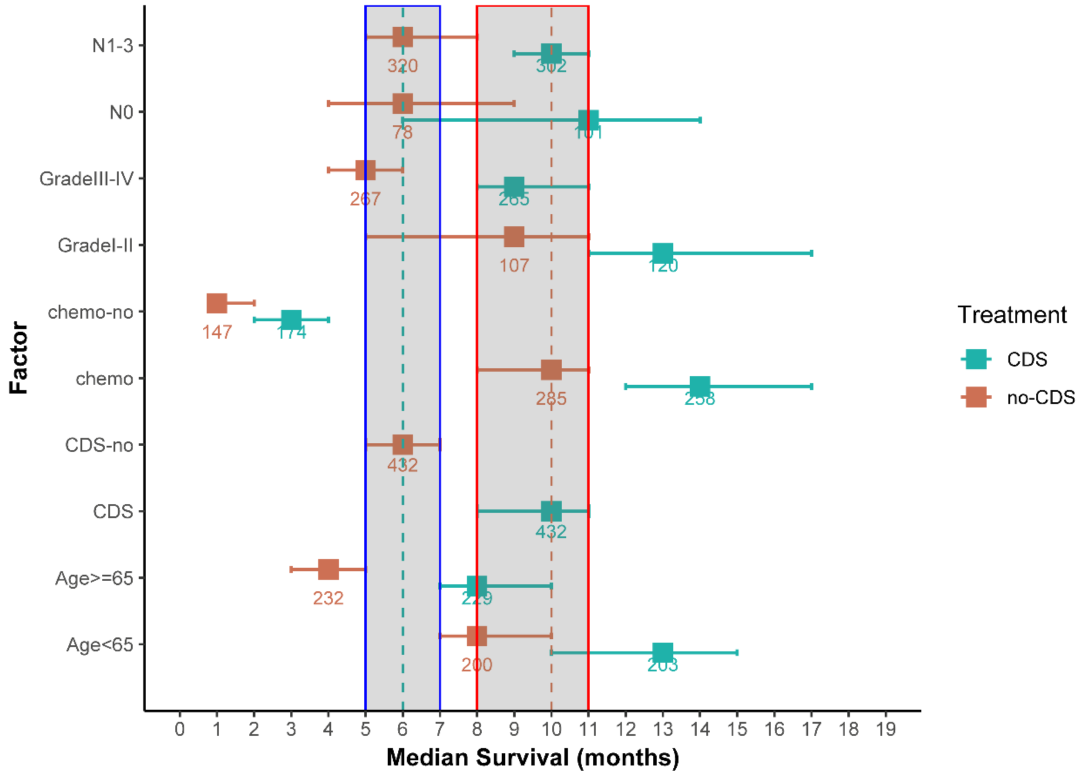

Figure 3. Kaplan-Meier overall survival (OS) estimates and 95% confidence intervals for patients in the CDS and no-CDS groups. (A) In the total sample, (B) Chemotherapy, (C) Grade, (D) N-stage, and (E) age. CDS: cancer-guided surgery.

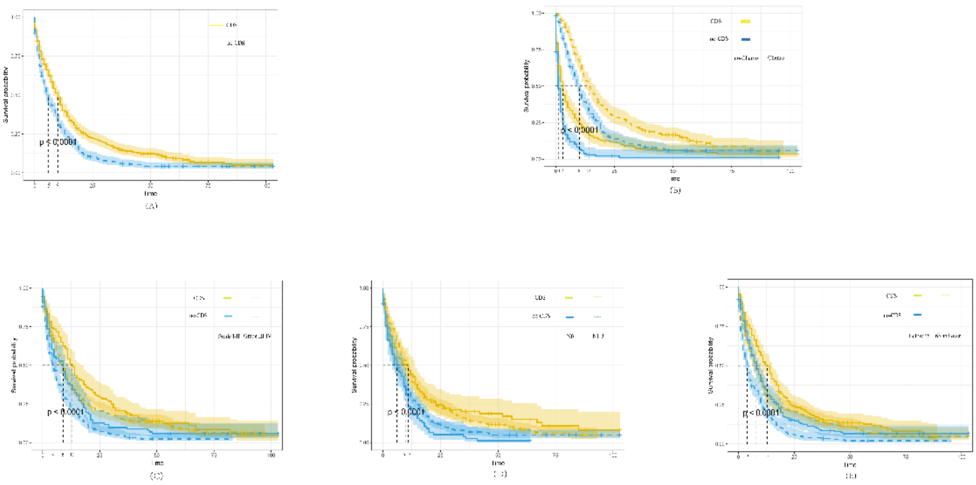

Figure 4. Full model average Cox proportional hazard ratios with 95% confidence intervals. There is a dashed line indicating the equivalent hazard ratio (HR=1) (Table 3).

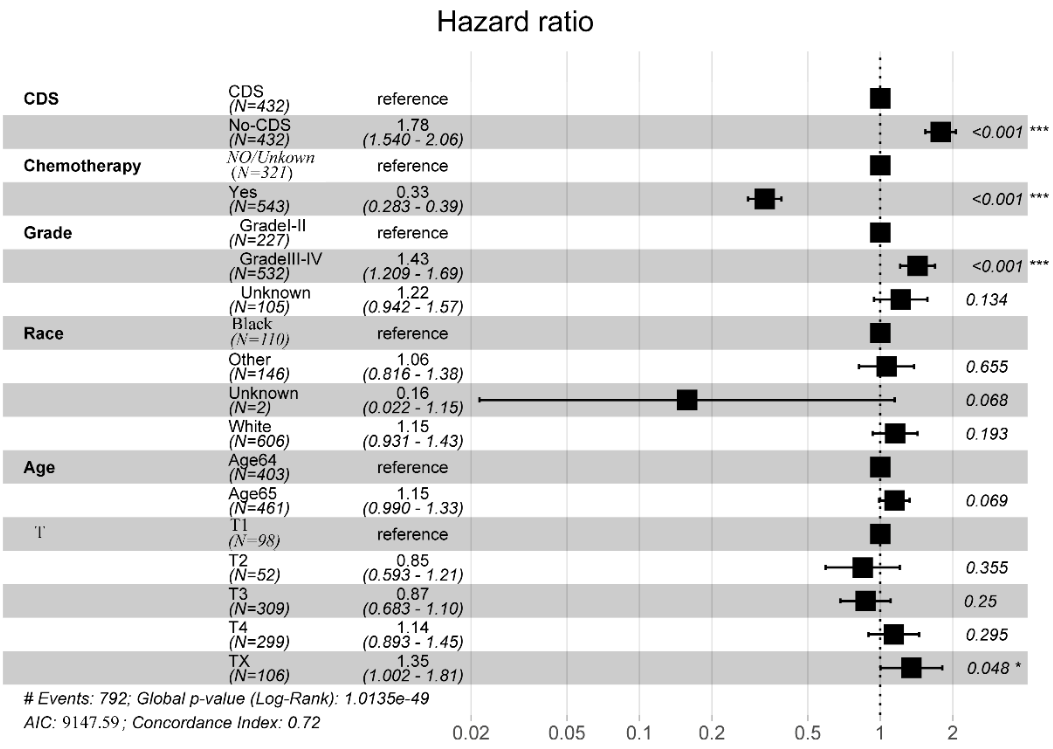

Supplement: Supplementary file 1 [file Datasheet1.pdf]
