# Supplementary figures and images for: Survival benefit of surgical resection for stage IV gastric cancer: A SEER-based propensity score-matched analysis
Source: Front Surg. 2022 Oct 25;9:927030. doi: 10.3389/fsurg.2022.927030 (PMC9640680; doi:10.3389/fsurg.2022.927030)

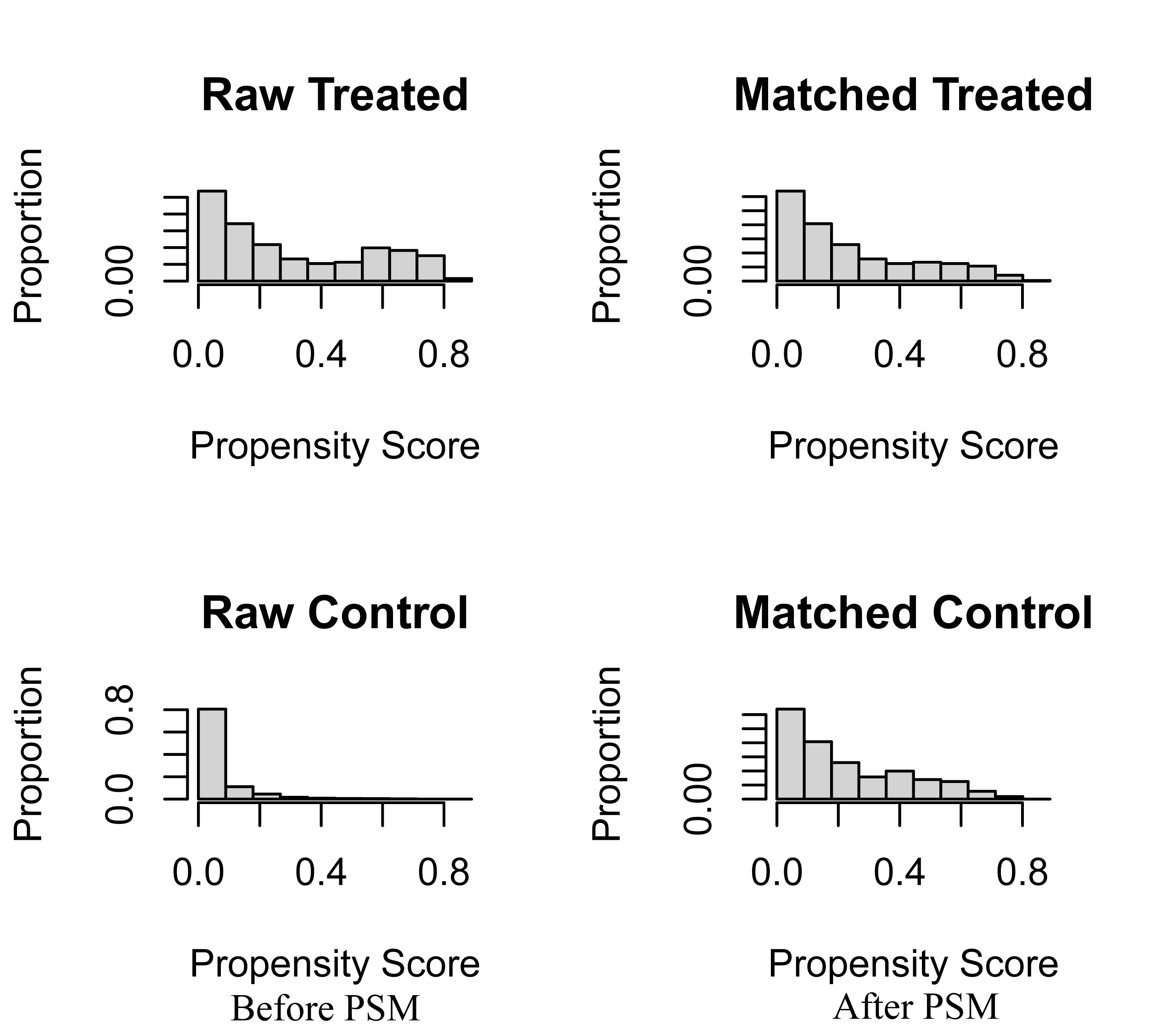

Supplement: Supplementary file 2 [file Datasheet2.zip › Figure(revised)/Figure 1.tif]

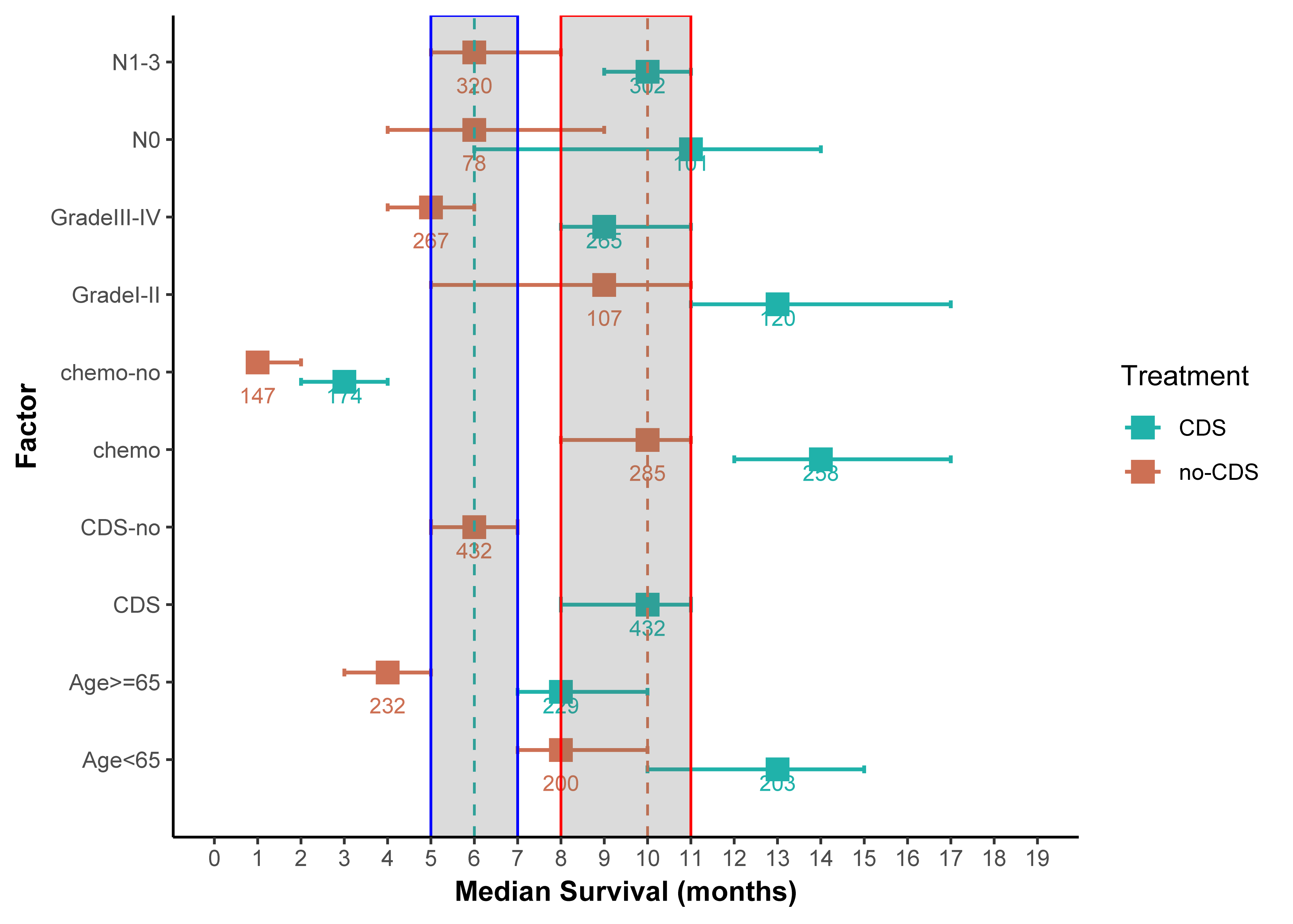

Supplement: Supplementary file 2 [file Datasheet2.zip › Figure(revised)/Figure 2.tif]

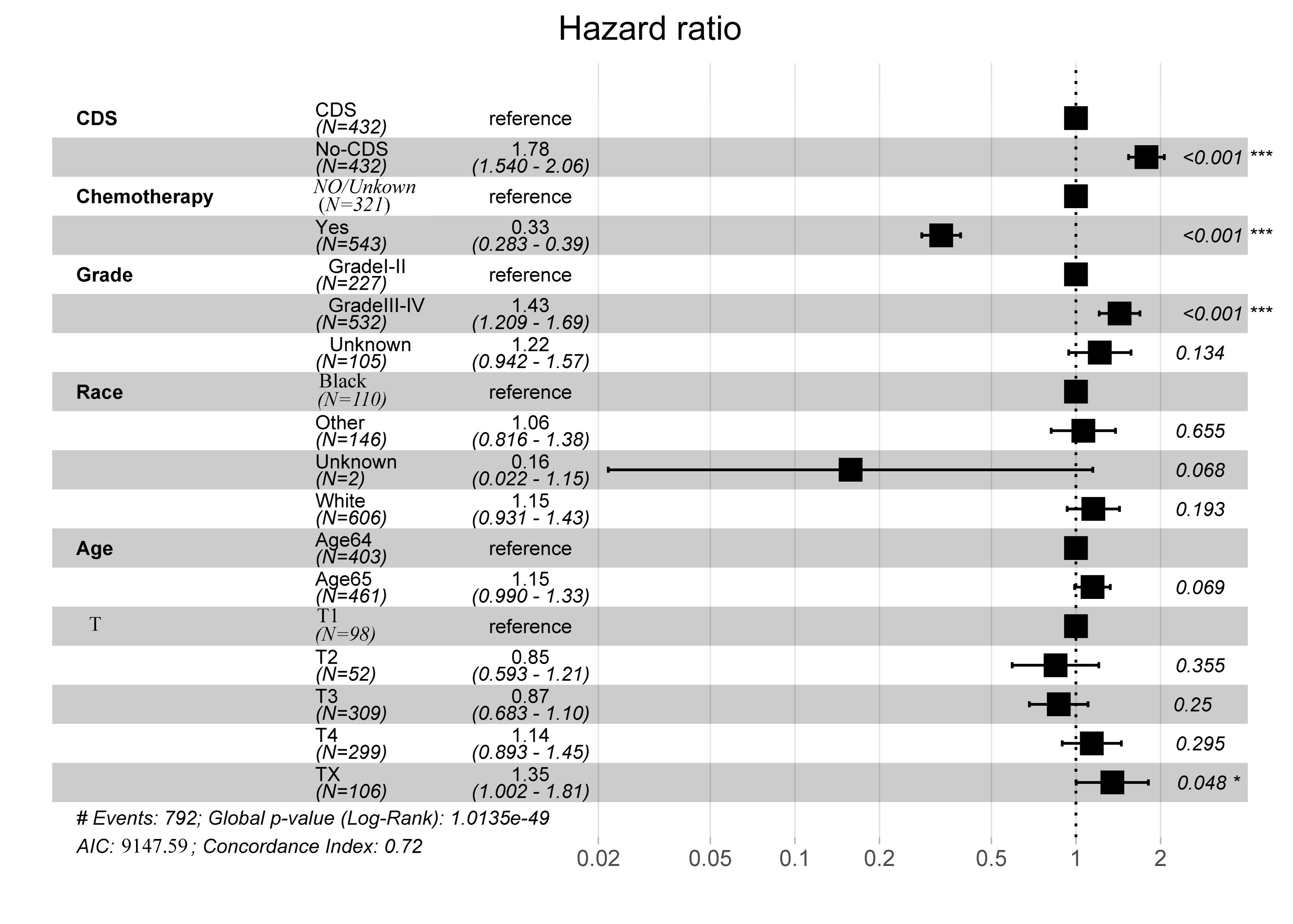

Supplement: Supplementary file 2 [file Datasheet2.zip › Figure(revised)/Figure 4.tif]
