# Supplementary material for: Survival benefit of surgical resection for stage IV gastric cancer: A SEER-based propensity score-matched analysis
Source: Front Surg. 2022 Oct 25;9:927030. doi: 10.3389/fsurg.2022.927030 (PMC9640680; doi:10.3389/fsurg.2022.927030)
Supplement: Supplementary file 3 [file Datasheet3.pdf]

Table 1. Baseline characteristics before propensity matching scores, showing statistical comparisons between the CDS and no-CDS groups.

	CDS (N=514)	No-CDS (N=5770)	Overall (N=6284)	χ^2	p
**Age**				3.521	0.172
≤49	61 (11.9%)	644 (11.2%)	705 (11.2%)		
50-64	186(36.2%)	1884(32.7%)	2070(32.9%)		
≥65	267 (51.9%)	3242 (56.2%)	3509 (55.8%)		
**Sex**				0.972	0.324
Female	165 (32.1%)	1726 (29.9%)	1891 (30.1%)		
Male	349 (67.9%)	4044 (70.1%)	4393 (69.9%)		
**Race**				26.668	<0.001
White	341 (66.3%)	4326 (75.0%)	4667 (74.3%)		
Black	71 (13.8%)	739 (12.8%)	810 (12.9%)		
Other	100 (19.5%)	687 (11.9%)	787 (12.5%)		
Unknown	2 (0.4%)	18 (0.3%)	20 (0.3%)		
**Marital status**				6.569	0.161
Divorced	41 (8.0%)	503 (8.7%)	544 (8.7%)		
Married	313 (60.9%)	3322 (57.6%)	3635 (57.8%)		
Single	61 (11.9%)	893 (15.5%)	954 (15.2%)		
Widowed	59 (11.5%)	680 (11.8%)	739 (11.8%)		
Unknown	40 (7.8%)	372 (6.4%)	412 (6.6%)		
**Primary Site**				148.950	<0.001
Body of stomach	40 (7.8%)	488 (8.5%)	528 (8.4%)		
Overlapping lesion of stomach	48 (9.3%)	378 (6.6%)	426 (6.8%)		
Stomach	75 (14.6%)	953 (16.5%)	1028 (16.4%)		
Cardia and fundus of stomach	140 (27.2%)	2756 (47.8%)	2896 (46.1%)		
Gastric antrum and pylorus	149 (29.0%)	730 (12.7%)	879 (14.0%)		
Greater and lesser curvature	62 (12.1%)	465 (8.1%)	527 (8.4%)		
**Grade**				69.774	<0.001
Grade I	4 (0.8%)	125 (2.2%)	129 (2.1%)		
Grade II	128 (24.9%)	1342 (23.3%)	1470 (23.4%)		
Grade III	318 (61.9%)	3046 (52.8%)	3364 (53.5%)		
Grade IV	17 (3.3%)	52 (0.9%)	69 (1.1%)		
Unknown	47 (9.1%)	1205 (20.9%)	1252 (19.9%)		
**T**				543.500	<0.001
T0	0 (0%)	33 (0.6%)	33 (0.5%)		
T1	48 (9.3%)	1185 (20.5%)	1233 (19.6%)		
T2	28 (5.4%)	174 (3.0%)	202 (3.2%)		
T3	173 (33.7%)	730 (12.7%)	903 (14.4%)		

T4	216 (42.0%)	866 (15.0%)	1082 (17.2%)		
Tx	49 (9.5%)	2782 (48.2%)	2831 (45.1%)		
**N**				849.840	<0.001
N0	101 (19.6%)	2036 (35.3%)	2137 (34.0%)		
N1	140 (27.2%)	2189 (37.9%)	2329 (37.1%)		
N2	110 (21.4%)	258 (4.5%)	368 (5.9%)		
N3	134 (26.1%)	170 (2.9%)	304 (4.8%)		
Nx	29 (5.6%)	1117 (19.4%)	1146 (18.2%)		
**Chemotherapy**				0.429	0.512
No	221 (43.0%)	2389 (41.4%)	2610 (41.5%)		
Yes	293 (57.0%)	3381 (58.6%)	3674 (58.5%)		
**Survival months**					
Mean (SD)	16.0 (19.4)	8.64 (12.6)	-	329.13	<0.001
Median [Min, Max]	9.00 [0, 103]	4.00 [0, 107]	-		

Table 2. Baseline characteristics after propensity matching scores, showing statistical comparisons between the CDS and no-CDS groups.

	**CDS (N=432)**	**No-CDS (N=432)**	**Overall (N=864)**	χ^2	**p**
**Age**				3.119	0.210
≤49	50 (11.6%)	35 (8.1%)	85 (9.8%)		
50-64	153 (35.4%)	165 (38.2%)	318(36.8%)		
≥65	229 (53.0%)	232 (53.7%)	461(53.4%)		
**Sex**				0.551	0.458
Female	135 (31.3%)	124 (28.7%)	259(30.0%)		
Male	297 (68.8%)	308 (71.3%)	605(70.0%)		
**Race**.				1.443	0.696
White	295 (68.3%)	311 (72.0%)	606(70.1%)		
Black	59 (13.7%)	51 (11.8%)	110(12.7%)		
Other	77 (17.8%)	69 (16.0%)	146(16.9%)		
Unknown	1 (0.2%)	1 (0.2%)	2 (0.2%)		
**Marital status.**				2.566	0.633
Divorced	31 (7.2%)	31 (7.2%)	62 (7.2%)		
Married	262 (60.6%)	266 (61.6%)	528(61.1%)		
Single	57 (13.2%)	43 (10.0%)	100(11.6%)		

Widowed	48 (11.1%)	54 (12.5%)	102(11.8%)		
Unknown	34 (7.9%)	38 (8.8%)	72 (8.3%)		
**Primary Site**				2.975	0.704
Body of stomach	36 (8.3%)	28 (6.5%)	64 (7.4%)		
Overlapping lesion of stomach	39 (9.0%)	36 (8.3%)	75 (8.7%)		
Stomach, NOS	65 (15.0%)	72 (16.7%)	137(15.9%)		
Cardia and fundus of stomach	139 (32.2%)	151 (35.0%)	290(33.6%)		
Gastric antrum and pylorus	97 (22.5%)	99 (22.9%)	196(22.7%)		
Greater and lesser curvature	56 (13.0%)	46 (10.6%)	102(11.8%)		
**Grade**				2.969	0.563
Grade I	4 (0.9%)	2 (0.5%)	6 (0.7%)		
Grade II	116 (26.9%)	105 (24.3%)	221(25.6%)		
Grade III	259 (60.0%)	258 (59.7%)	517(59.8%)		
Grade IV	6 (1.4%)	9 (2.1%)	15 (1.7%)		
Unknown	47 (10.9%)	58 (13.4%)	105(12.2%)		
**T**				4.818	0.307
T1	48 (11.1%)	50 (11.6%)	98 (11.3%)		
T2	28 (6.5%)	24 (5.6%)	52 (6.0%)		
T3	144 (33.3%)	165 (38.2%)	309(35.8%)		
T4	163 (37.7%)	136 (31.5%)	299(34.6%)		
Tx	49 (11.3%)	57 (13.2%)	106(12.3%)		
**N**				4.524	0.340
N0	101 (23.4%)	78 (18.1%)	179(20.7%)		
N1	140 (32.4%)	155 (35.9%)	295(34.1%)		
N2	88 (20.4%)	84 (19.4%)	172(19.9%)		
N3	74 (17.1%)	81 (18.8%)	155(17.9%)		
Nx	29 (6.7%)	34 (7.9%)	63 (7.3%)		
**Chemotherapy**				3.351	0.067
No	174 (40.3%)	147 (34.0%)	321(37.2%)		
Yes	258 (59.7%)	285 (66.0%)	543(62.8%)		
**Survival months**					
Mean (SD)	16.7 (19.7)	10.6 (15.2)		102.65	<0.001
Median [Min, Max]	10.0 [0, 103]	5.00 [0, 103]			

Table 3. Stepwise regression analysis method for automated model screening.

	HR	95%CI	P
**Age**			
<65	Reference		
≥65	1.14534	1.01315876-1.2947705	0.069
**Race**			
Black	Reference		
White	1.15323	0.96327929-1.3806284	0.193
Other	1.06197	0.85117918-1.3249732	0.655
Unknown	0.15777	0.02983461-0.8343466	0.068
**Grade**			
Grade I-II	Reference		
Grade III-IV	1.42907	1.24228609-1.6439408	<0.001
Unknown	1.21551	0.98108638-1.5059437	0.134
**T**			
T1	Reference		
T2	0.84571	0.62794846-1.1389890	0.356
T3	0.86839	0.70968537-1.0625911	0.250
T4	1.13828	0.92875278-1.3950809	0.295
Tx/NA	1.34726	1.05125127-1.7266097	0.048
**Chemotherapy**			
No	Reference		
Yes	0.33161	0.29021219-0.3789054	< 0.001
**Surgery**			
CDS	Reference		
no-CDS	1.78204	1.57616003-2.0148153	< 0.001

HR, Hazard ratio; CI, Confidence interval.
